# Modeling Effects of Temperature, Soil, Moisture, Nutrition and Variety As Determinants of Severity of Pythium Damping-Off and Root Disease in Subterranean Clover

**DOI:** 10.3389/fmicb.2017.02223

**Published:** 2017-11-14

**Authors:** Ming P. You, Kelly Rensing, Michael Renton, Martin J. Barbetti

**Affiliations:** ^1^UWA School of Agriculture and Environment, The UWA Institute of Agriculture, Faculty of Science, The University of Western Australia, Crawley, WA, Australia; ^2^Schools of Biological Sciences and Agriculture and Environment, The UWA Institute of Agriculture, Faculty of Science, The University of Western Australia, Crawley, WA, Australia

**Keywords:** environmental influence, soilborne root disease, damping-off, *Pythium irregulare*, subterranean clover, *Trifolium subterraneum*

## Abstract

Subterranean clover (*Trifolium subterraneum*) is a critical pasture legume in Mediterranean regions of southern Australia and elsewhere, including Mediterranean-type climatic regions in Africa, Asia, Australia, Europe, North America, and South America. Pythium damping-off and root disease caused by *Pythium irregulare* is a significant threat to subterranean clover in Australia and a study was conducted to define how environmental factors (viz. temperature, soil type, moisture and nutrition) as well as variety, influence the extent of damping-off and root disease as well as subterranean clover productivity under challenge by this pathogen. Relationships were statistically modeled using linear and generalized linear models and boosted regression trees. Modeling found complex relationships between explanatory variables and the extent of Pythium damping-off and root rot. Linear modeling identified high-level (4 or 5-way) significant interactions for each dependent variable (dry shoot and root weight, emergence, tap and lateral root disease index). Furthermore, all explanatory variables (temperature, soil, moisture, nutrition, variety) were found significant as part of some interaction within these models. A significant five-way interaction between all explanatory variables was found for both dry shoot and root dry weights, and a four way interaction between temperature, soil, moisture, and nutrition was found for both tap and lateral root disease index. A second approach to modeling using boosted regression trees provided support for and helped clarify the complex nature of the relationships found in linear models. All explanatory variables showed at least 5% relative influence on each of the five dependent variables. All models indicated differences due to soil type, with the sand-based soil having either higher weights, greater emergence, or lower disease indices; while lowest weights and less emergence, as well as higher disease indices, were found for loam soil and low temperature. There was more severe tap and lateral root rot disease in higher moisture situations.

## Introduction

Subterranean clover (*Trifolium subterraneum*) is critical pasture legume in Mediterranean regions of southern Australia and elsewhere, including Mediterranean-type climatic regions in Africa, Asia, Australia, Europe, North America, and South America (Nichols et al., 2014). In southern Australia, it has been grown on 29 million ha (Hill and Donald, 1998), where it provides critical nutrition as livestock feed and as a source of nitrogen for rotational cereal crops. However, subterranean clover is attacked by a diverse range of soilborne pathogens, especially oomycetes. Oomycete soilborne pathogens cause severe pre- and post-emergence damping off and root disease in seedlings (Wong et al., 1984; Barbetti et al., 1986a,b, 2006b, 2007) and root disease in subterranean clover on mature plants later in spring (O’Rourke et al., 2009), resulting in severe reduction of forage crop (Gillespie, 1983; Barbetti et al., 1986a). In-field losses in subterranean clover forages include up to 90% of seedlings from damping-off (Wong et al., 1985a), ≥70% productivity across the 6–17 weeks period post-germination (Barbetti, 1984a). Widespread deterioration of subterranean clover forages from damping-off and root disease seriously and adversely curtails both livestock carrying capacity *per se* and overall profitability (Barbetti et al., 1986b; Nichols et al., 2014), with some farmers no longer remaining economically viable in worst-affected areas.

Of the range of different soilborne pathogens occurring on subterranean clover, several *Pythium* species, particularly *P. irregulare, Phytophthora clandestina, Aphanomyces trifolii*, and *Rhizoctonia solani* (Barbetti and MacNish, 1978; Taylor et al., 1985a,b; Wong et al., 1985b; Barbetti et al., 2006a,b, 2007; You et al., 2006; Ma et al., 2008; O’Rourke et al., 2009, 2010; Nichols et al., 2014; You and Barbetti, 2017a) are considered the most important. Of these, *P. irregulare* in particular has been the most common pathogen isolated from diseased roots and reported as responsible for extensive pre-emergence damping-off and severe root rot in subterranean clover across southern Australia (Stovold, 1971, 1974a,b; Barbetti and MacNish, 1978; Greenhalgh and Lucas, 1984; Wong et al., 1984). You et al. (2005a) were the first to locate effective host resistance to *P. irregulare* in subterranean clover.

Several historical attempts have been made to relate one or more environmental influences to the severity of subterranean clover root disease. These include aspects of south coastal climatic data for Western Australia that highlighted an association of severe root disease with years of heavier and more frequent rainfall and lower disease severities in lower rainfall years (MacNish et al., 1976). In particular for *P. clandestina* in subterranean clover, there have been several studies involving the relationship of environmental factors with root disease severity. Examples include the behavior of this pathogen in the soil in the field (Wong et al., 1986b), the influence of environmental factors, including soil temperature and moisture, on pathogen growth, survival and level of root disease (Wong et al., 1986b,c; You and Barbetti, 2017b), and influence of nutrition on the expression of root disease (O’Rourke et al., 2012). However, there have been very few studies in relation to environmental effects specifically on the behavior of *P. irregulare*. Those few include Wong et al. (1984) who demonstrated that specific temperature/moisture combinations resulted in the lowest seedling survival (10°C/45% Water Holding Capacity [WHC]; 15°C/flooding), most severe root disease (flooding across 10, 15, 20, and 25°C) and smallest shoots (10°C/45%WHC; 15°C/flooding). However, in these individual studies on *P. irregulare* too few environmental factors were considered. Hence, controlled environment studies were undertaken to determine the discrete and interactive effects of what were considered to be the most likely overriding factors, viz. temperature, moisture, soil type, nutrition and subterranean clover variety as representative of subterranean clover forages across southern Australia, on Pythium damping-off and root disease caused by *P. irregulare*. Linear and generalized linear models and boosted regression trees were then utilized to elucidate the complex relationships and interactions amongst environmental conditions, variety and disease impacts.

## Materials and Methods

### Subterranean Clover Varieties, Temperature Regimes, Moisture Levels, Soil Types and Treatments

Varieties Riverina, Seaton Park and Woogenellup were used. Seaton Park is known to be highly susceptible to Pythium damping-off and root disease caused by *P. irregulare*, while Woogenellup is moderately susceptible and Riverina moderately resistant (Nichols et al., 2014). Woogenellup has been widely utilized as a standard susceptible control across nearly all historical subterranean clover soilborne root disease studies (Barbetti et al., 2006b, 2007). Similar as in previous studies of You and Barbetti (2017a,b), subterranean clover seeds were surface sterilized in 70% ethanol for 30 s to remove any pathogen seed contamination; then scarified lightly with sandpaper to remove dormancy and then sown at five seeds per pot at a depth of 10 mm in pots 9 cm × 9 cm square × 10 cm depth.

Experiments were conducted in a similar manner as used by You and Barbetti (2017a,b). Briefly, three separate controlled environment rooms were used, with temperatures maintained at 22/17°C or 18/13°C or 14/9°C (day/night) with a 12-h photoperiod and light intensity of 540 μM m^-2^ s^-1^. Temperatures selected mirror field temperatures in fields in Western Australia during the 1st months of the winter growing season from May onward to August, a time when particularly severe root disease most common in subterranean clover forages (Barbetti, 1991). These temperatures also approximate those used in earlier studies of subterranean clover soilborne diseases by Wong et al. (1984, 1986c).

Two levels of moisture were maintained, high moisture where pots were watered to free draining with deionized water each day [i.e., watered to 100% water holding capacity (WHC)]. For low moisture pots, and water was added by weight as needed to maintain 50% WHC as described by You and Barbetti (2017b).

Two types of soil, a sand-based mix representing light sand-based soil type (airing) and a Gingin red loam soil representing heavy soil type (compact), as described by You and Barbetti (2017b), were used. Briefly, sand-based soil consisted of 2.5 m^3^ fine composted pine bark, 1 m^3^ coco peat, 5 m^3^ brown washed river sand, 10 kg slow release fertilizer Osmoform^®^ NXT 22 N + 2.2 P_2_O_5_ + 9.1 K_2_O + 1.2 Mg + trace elements (Everris International B.V.), 10 kg Dolomite (CalMag^®^), 5 kg gypsum clay breaker, 5 kg extra fine limestone, 4 kg iron hepta sulfate, and 1 kg iron chelate). Gingin red was a loam soil with a sand content of 85% (w/w) (McArthur, 1991), representative of extensive soil areas in regions of southern Australia and this soil had no amendments. Both soils were pasteurized separately using aerated steam at 65°C for 90 min on each of three consecutive days prior to its use.

### Nutrition Levels and Soil Nutrient Analyses

Two levels of nutrition were utilized as described earlier in You and Barbetti (2017b); high nutrition where fertilized with a complete nutrient solution at the recommended rate weekly (Thrive, Yates^®^; N 25%, P5, K 8.8) and low nutrition treatment where seedlings were watered with only deionised water throughout. As described in You and Barbetti (2017b), 20 pots were used to make pooled soil samples collected from root zones around subterranean clover seedlings from each high or low treatment separately for sand-based and loam soils, and were air-dried at 25–30°C in a glasshouse, and nutrient analyses undertaken by CSBP Plant and Soil Analysis Ltd., Western Australia. A summary of soil nutrient analyses as detailed in You and Barbetti (2017b) is as follows: In comparison with the Gingin red loam, sand-based soil under high nutrient treatment contained higher nitrate Nitrogen (15.56 mg kg^-1^), Phosphorus (Colwell 39.67 mg kg^-1^), Potassium (Colwell, 79.72 mg kg^-1^), Sulfur (28.8 mg kg^-1^), Organic Carbon (4.22%), Conductivity (0.11 dS m^-1^), Copper (0.71 mg kg^-1^), Iron (17.27 mg kg^-1^), Zinc (1.65 mg kg^-1^), Potassium (89.33 meq 100g^-1^), Sodium (0.07 meq 100g^-1^), Boron (0.38 mg kg^-1^), Nitrogen (Total, 0.09%), Phosphorus (Total, 79.95 mg kg^-1^) and Potassium (Total, 89.33 mg kg^-1^). Under low nutrition treatment, in comparison with the Gingin red loam, the sand-base soil contained the highest levels of Manganese (2.34 mg kg^-1^), Calcium (6.321 meq 100 g^-1^), and Exc. Magnesium (2.34 meq 100 g^-1^). Exceptions to the above were where Gingin red loam under high nutrition treatment contained higher levels of ammonium Nitrogen (11.06 mg kg^-1^) and under both low and high nutrition contained higher Aluminum (0.06 and 0.05 meq 100 g^-1^, respectively) in comparison with the sand-based soil.

### Inoculum Production of *P. irregulare* and Inoculation, Confirmation of *P. irregulare* Presence

A single isolate of *P. irregulare* was used, viz. WAC4953, from the Western Australian Culture Collection, Department of Agriculture and Food Western Australia. The isolate was originally from subterranean clover and had been widely utilized for studies into damping-off and/or root rots in subterranean clover (e.g., You et al., 2005a) and different legumes (e.g., Li et al., 2015) and well represents *P. irregulare* populations in Western Australia (Li et al., 2015).

Inoculum was prepared as described by Li et al. (2015) as follows: *P. irregulare* was cultured on potato dextrose agar (PDA) for 5 days at 25°C in the dark until mycelium had almost grown across the plate. Then, sterilized millet seed produced as detailed in You and Barbetti (2017b) ‘seeded’ with five *P. irregulare*-colonized agar pieces and incubated at 25°C in the dark for 2 weeks.

One half of each soil type was mixed thoroughly with *P. irregulare*-colonized millet seeds at a rate of 0.5% (w/w) immediately prior to sowing and used to fill pots (You and Barbetti, 2017b). The control treatment for comparison was pots containing uninfested soil of each type, but without any uncolonised millet seeds added as uncolonised millet can readily ‘bait-out’ other non-target soil-borne pathogens present (Barbetti and Sivasithamparam, 1987; You and Barbetti, 2017b). That only *P. irregulare* was present in the inoculum, was confirmed by plating 15 colonized millet seeds onto corn meal agar (Sigma–Aldrich Chemie GmbH, Buchs, Switzerland) plates (Li et al., 2015). In each experiment, 200 g of inoculated soil was placed in each pot (Li et al., 2015).

In all experiments Koch’s postulates were successfully completed as described in Li et al. (2015) to confirm that the disease symptoms observed were in fact caused by the *P. irregulare* as follows: Root segments (8–10), 2 cm in length, were dissected from diseased plants and floated in Petri dishes containing sterile deionised water for 2–3 days at 20°C. Five roots from each treatment were examined microscopically every 12 h using a light microscope and the presence of *P. irregulare* zoosporangia confirmed.

### Disease and Plant Weight Assessments

Disease and plant weight assessments were as earlier described by You and Barbetti (2017b) as follows: Briefly, germinated plants in each pot were counted to calculate emergence percentage before harvesting. Then, plants were harvested at 5 weeks after sowing, washed in running tap water to remove soil from roots and scored for their level of root disease. Plants were then floated in shallow trays of deionised water and both tap and lateral roots were visually scored independently using a modified scoring system described and used earlier by Wong et al. (1984); where score 0 = root healthy, no discolouration; 1 = <25% of root brown, no significant lesions; 2 = 25–<50% of root brown, lesions toward base of tap root; 3 = 50–<75% root brown, lesions mid tap root; 4 = ≥75% root affected, significant lesions toward crown; 5 = plant dead and/or root system completely rotted off. The number of plants in each disease severity category was recorded. Then, all disease rating scores were transferred to a tap (TDI) or lateral (LDI) “Percent Disease Index” based on (McKinney, 1923) and as detailed in You and Barbetti (2017b).

Shoots and roots from each pot were separated and dried at 60°C in an oven in separate paper bags for 3 days then dry shoot and root weights were recorded and calculated as mg plant^-1^.

### Experimental Design, Modeling Approach and Data Analyses

Experimental design was as described earlier by You et al. (2017) as follows: There were four replicate pots for each treatment, with treatments in a full factorial arrangement, and all pots were maintained in their respective temperature-controlled environmental rooms throughout. All inoculated treatments were repeated using non-inoculated soils as control comparisons. This experiment was arranged in a randomized complete block design and the whole experiment for inoculated and uninoculated soils was fully repeated once under the same conditions. Data from the original and the repeat experiments were not significantly different (*P* > 0.05) using a pairwise *t*-test. Therefore, data from the original and repeat experiments were combined and re-analyzed together.

Experimental data were analyzed using classical linear models (ANOVA) and generalized linear models (GLMs) to investigate the relationships between explanatory variables and the effects of Pythium damping-off and root disease on the three subterranean clover varieties. As these indicated complex and difficult to interpret interaction effects between explanatory variables, we also analyzed the data using boosted regression trees (BRTs), a complementary method that is well-suited to data with complex interactions, and can be summarized in ways that give powerful biological insight (Elith et al., 2008; James et al., 2013). Simple regression trees and heat maps were also employed to help visualize the complex interactions within the data. Separate analyses and visualizations were conducted for each of the five measured dependent variables, dry shoot and root weights, emergence, and tap and lateral root disease indices for inoculated plants. For dry shoot and root weights, if seeds germinated and visible roots were present prior to drying, but weights were below the instrument detection limit, then a weight of 50% of the minimum weight was assigned. Differences between inoculated and control plants were also calculated for dry shoot and root weights and emergence, based on the mean of the replicates within each given treatment. Calculating differences between treatment means meant there was no replication within treatment, and so difference data were only analyzed using BRTs as fitting of high order interactions within linear models, or GLMs would not have been possible. All analyses and data manipulations were conducted using the statistical package R (R Development Core Team, 2016), and its packages ‘dismo’ for boosted regression trees (Hijmans et al., 2016), ‘plotly’ for heat maps (Sievert et al., 2016) and ‘rpart’ and ‘rpart.plot’ for simple regression trees (Milborrow, 2015; Therneau et al., 2015).

Linear modeling was conducted on data from inoculated plants only. Linear models using boxcox power transformation were fitted for dependent variables dry shoot and root weights, while GLMs with binomial/quasibinomial error distribution and logit link function were performed for emergence, TDI and LDI. To accommodate zeros in the dataset, one was added to all dry shoot and root weight values prior to transformation. Initial models were fitted including all possible interactions, and simplified based on backward selection using *F*-test (linear models) or Chi-squared tests (GLMs) starting with the highest interaction term (Crawley, 2013). This means that highest order interaction terms were removed from the model unless the test showed the existing model was significantly better than the model with the term removed (*P*-value < 0.05). Diagnostic plots were examined to check model assumptions including homoscedasticity and normality of residuals.

Boosted regression trees were constructed for the inoculated plants data set and also on the calculated differences between inoculated and control plants, following the approach recommended by Elith et al. (2008). For all BRTs, model parameters were set as: family ‘Gaussian,’ tree complexity 5, learning rate 0.01, and bag fraction 0.5. These parameters allowed for a minimum of 1000 trees and maximized the model performance (lowest root mean squared error, RMSE). No terms were dropped from models. For dependent variables dry shoot and root weights, the same power transformation determined from linear modeling was used on the data prior to modeling.

For inoculated plants, BRT models were developed on the full dataset, as well as by cross-validation using training and test subsets of the data. For the latter, models were constructed using 75% of data and tested on the remaining 25%. Data were divided by replication number to provide balanced subsets (all possible explanatory variable combinations) and cross-validation was performed four times, separating a different replicate number out for testing each time. Root mean squared prediction error (RMSE) from the four cross-validations were then averaged, providing a more realistic measure of model performance than obtained from models fitted to the full data set, which predict values for the same data used to create models.

To help visualize any complex interactions between explanatory variables, regression trees were also constructed. As the purpose here was purely visualization, trees were grown and presented without pruning despite potential overfitting. Heat maps were created to further illustrate and examine relationships, as well as compare model predictions to actual data, using R package ‘plotly’ (Sievert et al., 2016).

## Results

### Overview and Outcomes from Modeling Approaches

Modeling found significant but complex relationships between explanatory variables and the presence of Pythium damping-off and root disease. Linear modeling identified high-level (4 or 5-way) significant interactions between explanatory variables for each dependent variable (dry shoot weight, dry root weight, emergence, tap root disease index, and lateral root disease index). Furthermore, all explanatory variables (temperature, soil, moisture, nutrition, variety) were found significant as part of some interaction within these models. The five-way interaction between all explanatory variables was significant when explaining both dry shoot and root weight, and a four way interaction between temperature, soil, moisture, and nutrition was significant when explaining both tap root disease index and lateral root disease index (**Table 1**).

**Table 1:** Significant interactions from linear and generalized linear modeling of the effects of environmental explanatory variables (moisture, temperature, nutrition, soil type, and variety) on the dependent variables dried shoot weight (DSW), dried root weight (DRW), emergence, and tap root and lateral root disease indices (TDI, LDI) of three subterranean clover (*Trifolium subterraneum*) varieties inoculated with *Pythium irregulare* in a controlled environment experiment.

Model	Significant interactions	df	*P*-value	Res. dev.	*R*^2^	RMSE
DSW	Temp × soil × moisture × nutrition × variety	4	0.002	–	0.66	11.17
DRW	Temp × soil × moisture x nutrition x variety	4	0.001	–	0.61	11.58
Emergence	Temp × moisture × nutrition × variety Temp × soil × variety	4 4	0.008 >0.001	304.33 on 216	–	2.49
TDI	Soil × moisture × nutrition × variety Temp × soil × moisture × nutrition	2 2	0.04 0.04	33.106 on 175	–	16.28
LDI	Temp × soil × moisture × nutrition Soil × variety	2 2	0.001 0.003	30.228 on 175	–	15.50

The second approach to modeling using boosted regression trees provided support for the complex nature of the relationships found in linear models. All explanatory variables showed at least 5% relative effect on each of the five dependent variables for both diseased plants as for the differences between diseased and control plants (**Table 2**).

**Table 2:** Percent variable relative influence from boosted regression tree modeling of the effects of environmental explanatory variables (moisture, temperature, nutrition, soil type, and variety) on the dependent variables dried shoot weight (DSW), dried root weight (DRW), emergence, and tap root and lateral root disease indices (TDI, LDI) of three subterranean clover (*Trifolium subterraneum*) varieties inoculated with *Pythium irregulare* in a controlled experiment.

	Variable relative influence (%)						
Model	Soil	Temp	Variety	Nutrition	Moisture	RMSE	Avg. test RMSE
**Diseased Plants**					
DSW	40.65	21.20	12.16	16.64	9.35	1.91	2.27
DRW	30.69	20.54	12.92	19.28	16.57	1.58	2.18
Emergence	50.50	12.54	17.87	10.64	8.47	1.08	1.17
TDI	28.81	21.11	24.44	7.42	18.22	0.03	0.04
LDI	19.65	21.09	23.66	9.42	26.18	0.03	0.04
**Differences in mean values for diseased and control plants**					
DSW	21.39	17.52	13.44	43.21	4.44	–	–
DRW	15.73	43.10	8.62	27.10	5.45	–	–
Emergence	52.43	26.30	7.78	8.32	5.17	–	–

**FIGURE 1:**
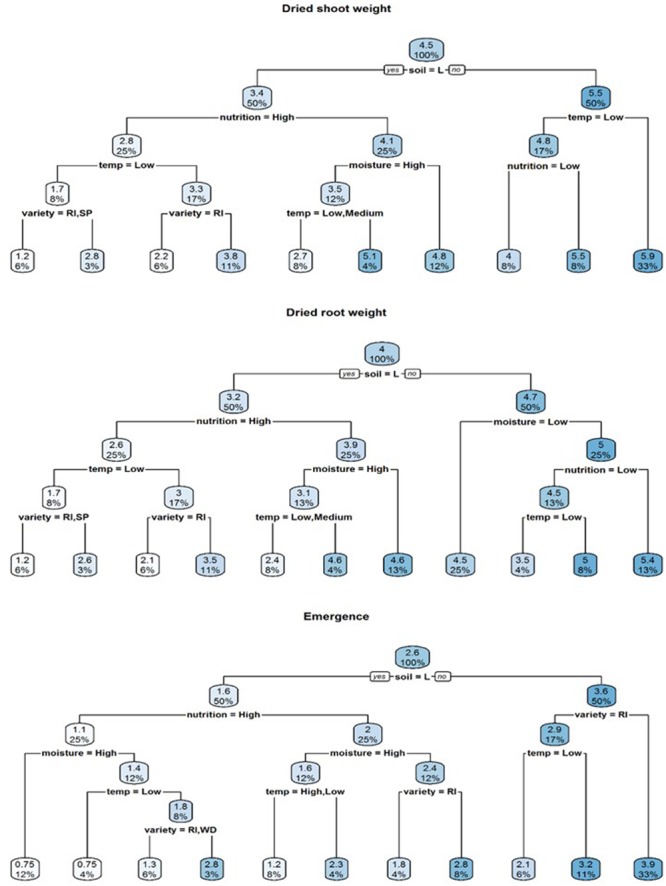
Regression decision trees illustrating the influence of environmental explanatory variables (moisture, temperature, nutrition, soil type, and variety) on the dependent variables dried shoot weight, dried root weight and emergence of three subterranean clover (*Trifolium subterraneum*) varieties inoculated with *Pythium irregulare* in a controlled environment experiment and grown under combinations of high (H) and low (L) moisture and nutrition and in sand-based soil (SBS) or loam soil (Loam). The numbers and the shading in the boxes represent the mean value at each decision point; the percentages indicate the percentage of all values considered at that decision point.

**FIGURE 2:**
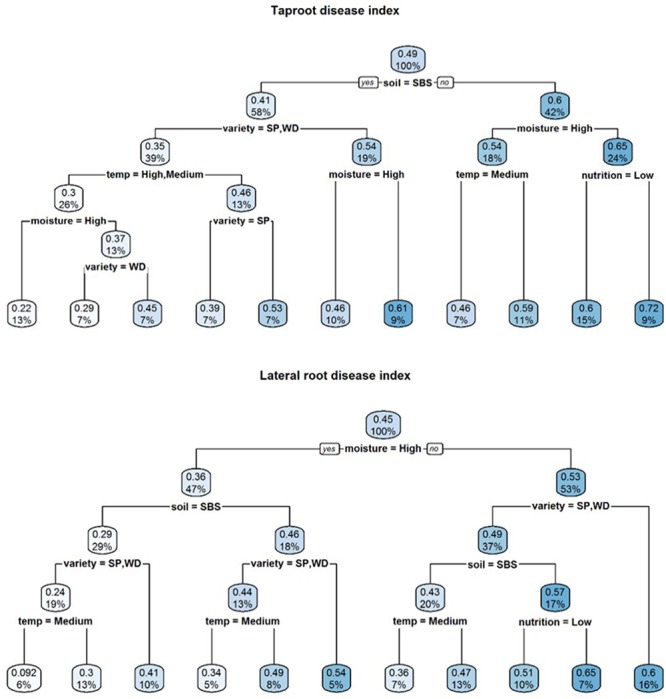
Regression decision trees illustrating the influence of environmental explanatory variables (moisture, temperature, nutrition, soil type, and variety) on the dependent variables tap root and lateral root disease indices (TDI, LDI) of three subterranean clover (*Trifolium subterraneum*) varieties inoculated with *Pythium irregulare* in a controlled environment experiment and grown under combinations of high (H) and low (L) moisture and nutrition and in SBS or loam soil (Loam). The numbers and the shading in the boxes represent the mean value at each decision point; the percentages indicate the percentage of all values considered at that decision point.

**FIGURE 3:**
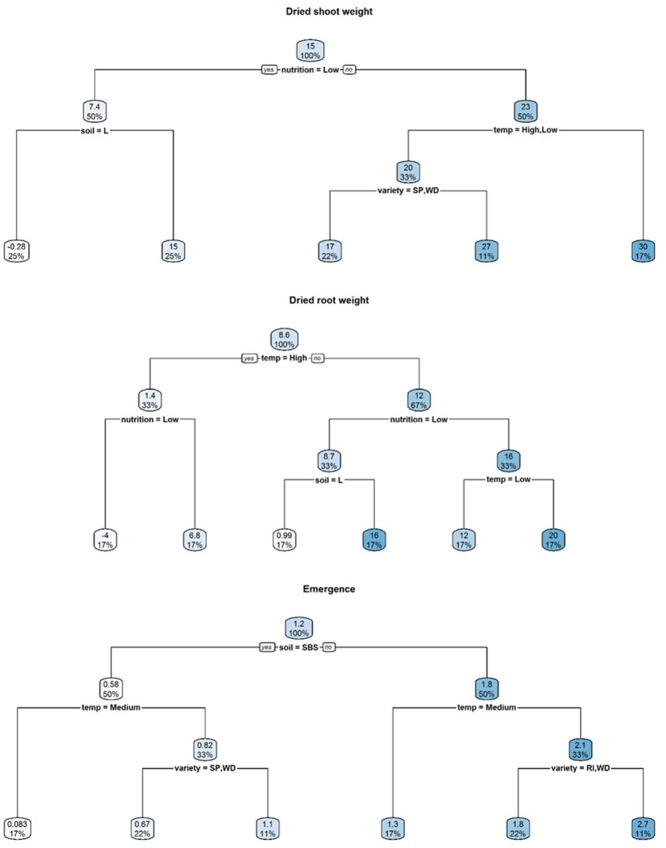
Regression decision trees illustrating the influence of environmental explanatory variables (moisture, temperature, nutrition, soil type, and variety) on the difference between mean dried shoot weight, dried root weight and emergence of three subterranean clover (*Trifolium subterraneum*) varieties inoculated with *Pythium irregulare* or control plants in a controlled environment experiment and grown under combinations of high (H) and low (L) moisture and nutrition and in SBS or loam soil (Loam). The numbers and the shading in the boxes represent the mean value at each decision point; the percentages indicate the percentage of all values considered at that decision point.

**FIGURE 4:**
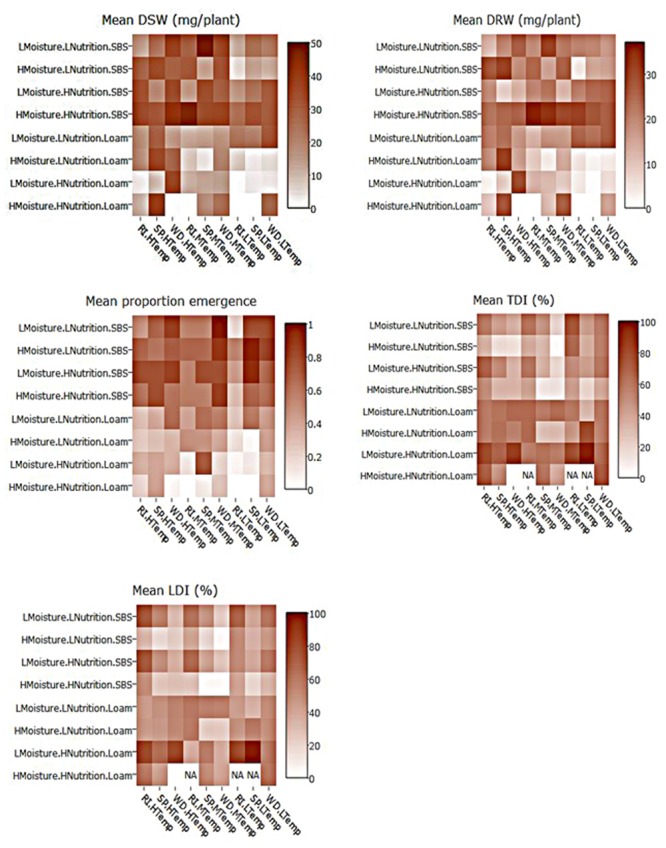
Heat maps of mean dry shoot weight (DSW), dry root weight (DRW), emergence, and tap and lateral root disease indices (TDI, LDI) of three subterranean clover varieties (*Trifolium subterraneum*) inoculated with *Pythium irregulare* in a controlled environment experiment and grown under combinations of high (H) and low (L) moisture and nutrition and in SBS or loam soil (Loam). Darker shading indicates higher values, as per the color scale bar. NAs values indicate missing data. On *X*-axis, RI, Riverina; SP, Seaton Park; and WD, Woogenellup varieties of subterranean clover and LTemp, MTemp, and HTemp are low, medium and high temperature regimes, respectively.

A visual representation of the complex relationships identified in linear and boosted regression tree modeling is provided in **Figures 1–3** and unpruned decision trees for inoculated plant data is provided in **Figures 1, 2**. All inoculated plant decision trees, aside from the tree for lateral root disease index, initially split with the factor soil. This was consistent with outputs from boosted regression trees which showed soil to have the highest relative effect (**Table 2**). For difference data, all models initially split with factors other than those found most influential in boosted regression trees (**Figure 4**).

**FIGURE 5:**
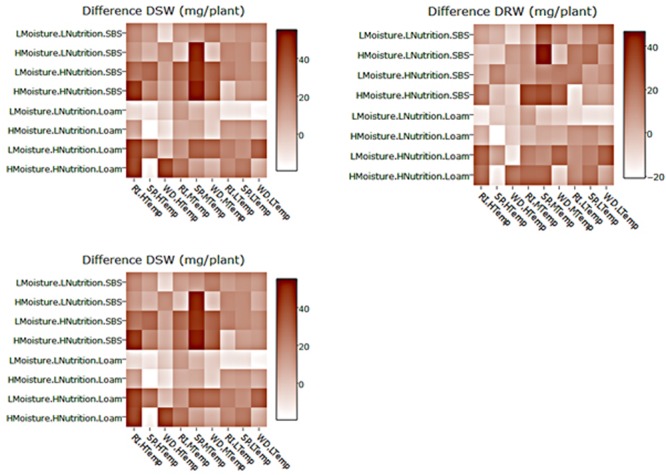
Heat maps of the differences between mean DSW, DRW and emergence for three subterranean clover (*Trifolium subterraneum*) varieties inoculated with *Pythium irregulare* or control plants in a controlled experiment and grown under combinations of high (H) and low (L) moisture and nutrition and in sand-based soil (SBS) or loam soil (Loam). Darker shading indicates higher values, as per the color scale bar. On *X*-axis, RI, Riverina; SP, Seaton Park; and WD, Woogenellup varieties of subterranean clover and LTemp, MTemp, and HTemp are low, medium, and high temperature regimes, respectively.

**FIGURE 6:**
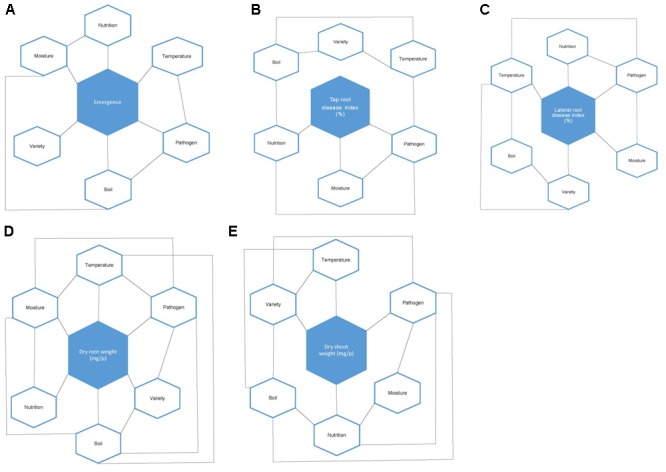
**(A–E)** Effect of environment explanatory factors (moisture, temperature, nutrition, soil type, and variety) and their interactions on: **(A)** subterranean clover (*Trifolium subterraneum*) emergence rate (%), **(B)** tap root disease index (%), **(C)** lateral root disease index (%), **(D)** dry root weight (mg/p), and **(E)** dry shoot weight (mg/p) in the presence of the soilborne pathogen *Pythium irregulare*. Hexagons simply represent the different explanatory variables.

Heat maps illustrating the mean actual values across explanatory variable combinations for inoculated plant data and the differences between inoculated and control are shown in **Figures 4, 5**. For all models, soil type was important, with plants in sand-based soils having either higher weights, greater emergence, or lower disease indices. Lowest weights and proportion emergence, as well as higher disease indices were found for loam soil and low temperature. In sand based soils, the greatest differences between inoculated and control plants were seen in high moisture conditions, while in loam soils, the greatest differences were seen in high nutrition conditions, although these relationships were somewhat obscured by interactions with variety and temperature (**Figure 5**). Boosted regression trees on the difference data showed that the greatest differences in root and shoot weights occurred in high nutrition, medium temperature conditions, especially on sand-based soils (Supplementary Figure 3). They also showed that the greatest differences in emergence occurred in low and high temperatures in loam soils (Supplementary Figure 3). Heat maps showing the predicted values from linear modeling and boosted regression trees illustrate the complexity of the interactions between the explanatory variables for all dependent variables (Supplementary Figures 1, 2). An overview of relationships between factors (moisture, temperature, nutrition, soil type, and variety) and their interactions influencing emergence, tap root disease index, lateral root disease index, dry root weight, and dry shoot weight in the presence of *P. irregulare* is illustrated in **Figures 6A–E**.

### Explanatory Variables – Dry Shoot Weight and Dry Root Weight

Modeling revealed a complex relationship between dry shoot weights of inoculated plants with Pythium damping-off and root disease and environmental factors. Linear modeling found significant 5-way interactions between all explanatory variables for both dry shoot and root weights (**Table 1**). BRTs provided further support for the effect of all explanatory variables on differences in dry root weights (**Table 2**). Relative effect of explanatory variables in BRT models was consistent for both dry shoot and root weights, with soil the most influential factor, followed by temperature, nutrition, variety and moisture.

Soil was also the initial splitting factor for both dry shoot and root weight decision trees (**Figure 2**). In these models, plants with the highest dry shoot weight were grown under conditions of sand-based soil, high or medium temperatures, while plants with the highest dry root weight were grown under conditions of sand-based soil, high moisture and high nutrition. The lowest dry shoot and root weights were varieties Riverina and Seaton Park, grown under the conditions of loam soil with high nutrition and low temperature.

Heat maps also provided a visual representation of the relationships showing a clear increase in dry shoot weight for plants grown under the conditions of sand-based soil and medium or high temperatures. A similar pattern was evident for dry root weight, but less clear. Lowest dry shoot and root weight were found from loam soils, particularly under low temperatures (**Figure 4**).

Results of modeling the differences between mean emergence of inoculated plants and control plants showed that temperature had the strongest relative influence on dry root weight (**Table 2**) and neither decision tree split initially with soil (**Figure 3**).

### Explanatory Variables – Emergence

Linear modeling identified two significant interactions for explaining the relationship between emergence and explanatory variables, a 3-way interactions between temperature, soil and variety, and a 4-way interactions between temperature, moisture, nutrition and variety (**Table 1**). Relative effect of these factors from BRT modeling found soil had a 50% effect on emergence, followed by variety, temperature, nutrition, and moisture (**Table 2**).

Decision trees found highest emergence were for varieties Woogenellup and Seaton Park grown in sand-based soil, while the lowest emergence proportions were from plants grown in loam soil, high nutrition and high moisture (**Figure 2**). Heat maps also showed higher emergence proportions for plants grown in sand-based soil rather than loam soil (**Figure 4**).

Results of modeling for the differences between mean emergence of inoculated plants and control plants showed a strong effect from soil type (**Table 2** and **Figure 3**) with larger differences in loam soil (**Figure 5**) but less clear for other factors.

### Explanatory Variables – Tap Root and Lateral Root Disease Index

Tap root disease index were found significantly affected by two 4-way interactions between temperature, soil, moisture and nutrition, and also interactions between soil, moisture, nutrition and variety. Lateral root disease index were found significantly affected by one 4-way and one 2-way interactions which were the interactions between temperature, soil, moisture and nutrition (same as for tap root disease index) and the interactions between soil and variety (**Table 1**). Boosted regression trees found similar relative effect on both tap and lateral root disease indices from soil, temperature and variety (**Table 2**).

In decision trees, the lowest disease indices were for Seaton Park and Woogenellup varieties grown in sand-based soil under high and medium temperatures and high moisture for tap root disease index. A similar pattern was found for lateral root disease index, but with a slightly different splitting pattern. Varieties Seaton Park and Woogenellup grown in sand-based soil under high moisture and medium temperature had the lowest disease indexes. The highest levels of root disease index were found from the conditions of loam soil, low moisture and high nutrition for tap roots and low moisture for lateral roots of variety Riverina (**Figure 3**).

Heat maps showed higher disease indices for plants grown on loam soil; however, this pattern was more apparent in tap root disease index than lateral root disease index. There was also a pattern for both tap and lateral root with higher levels of disease found in plants under high moisture (**Figure 4**).

## Discussion

These are the first studies to use a comprehensive modeling approach to highlight the importance of environmental conditions, as occur across southern Australia in upon the severity of Pythium damping-off and root disease and subterranean clover forage productivity. All explanatory variables (temperature, soil, moisture, nutrition, and variety) significantly affected severity of pre-emergence damping-off (i.e., emergence), root disease and root and shoot productivity and all environmental factors were found significant as part of some interaction within these models. Relationships between environmental factors and the presence of Pythium damping-off and root disease were complex, with linear modeling identifying high-level (4 or 5-way) significant interactions for each dependent variable (dry shoot and root weight, emergence, tap and lateral root disease index). For example, a significant five-way interaction between all factors was found on both dry shoot and root weights, and a four way interaction between temperature, soil, moisture, and nutrition was found on both tap and lateral root disease indices. A second approach to modeling using boosted regression trees provided support for the complex nature of the relationships found in linear models, with all explanatory variables showing at least 5% relative effect on each of the five dependent variables (temperature, soil, moisture, nutrition, and variety). For all models a clear trend was the difference in soil type, with the sand-based soil having either higher weights, greater emergence, or lower disease indices; while lowest weights and less emergence, as well as higher disease indices were for loam soil and low temperature.

Specifically in relation to emergence, linear modeling highlighted significant interactions between temperature, soil and variety, and between temperature, moisture, nutrition and variety. Relative influence of these explanatory variables from BRT modeling showed a 50% influence of soil type on emergence, followed by variety, temperature, nutrition, and moisture. When Wong et al. (1984) examined the influence of temperature and moisture on severity of damping-off caused by *P. irregulare*, they similarly found that specific combinations of multiple explanatory variables resulted in the greatest pre-emergence damping-off. For example, they found that temperature/moisture combinations of 10°C + 45%WHC or 15°C + flooding caused greatest pre-emergence damping-off. Similarly, for another oomycete pathogen, *P. clandestina*, You and Barbetti (2017b) showed significant interactions involving temperature, moisture, variety and soil type in terms of emergence, with cultivar resistance, high moisture, high or medium temperature, high nutrition and sand soil all contributing toward less pre-emergence damping-off and tap and lateral root disease and to greater clover productivity. That decision trees showed highest emergence for Woogenellup and Seaton Park grown in sand-based soil, but lowest emergence in loam, in high nutrition and high moisture is not surprising as Seaton Park is highly susceptible and Woogenellup moderately susceptible to damping-off by *P. irregulare* (Nichols et al., 2014). However, while Riverina is overall considered moderately resistant to root disease *per se* (Nichols et al., 2014), it showed poorest emergence, in line with field observations that in soilborne disease-conducive situations this latter variety is extremely susceptible to pre-emergence damping-off with consequent very poor emergence (You and Barbetti, unpublished data).

Heat maps and modeling differences between mean emergence of diseased plants and control plants demonstrated and confirmed the strong influence from soil type with less pre-emergence damping-off in sand-based soil vs. loam soil, similar as demonstrated with *P. clandestina* (You and Barbetti, 2017b). While *P. irregulare* is a serious pathogen across diverse soil types throughout southern Australia, from coarse sand to heavier loam or even clay based soils, it is a particularly devastating pre-emergence pathogen across the widely prevalent, impoverished and nutrient-deficient soils across south west of Western Australia (Barbetti et al., 2006b), soils that predispose plants to soilborne pathogens, particularly as microbial competition is minimal in such soils (Sivasithamparam, 1993). There, field losses of seedlings from damping-off can be >90% where *P. irregulare* dominates (Wong et al., 1985a) and the current study confirms that the importance of *P. irregulare* as a cause of extensive pre-emergence damping-off in subterranean clover across southern Australia (Barbetti and MacNish, 1978; Greenhalgh and Lucas, 1984; Wong et al., 1984, 1985a, 1986a,b). It is possible that the ‘disturbed’ soil seedbed structure in the current study could have resulted in an overestimation of emergence in comparison with the natural seedbed structures of field soils *in situ*. However, despite this, it still remains that any impedance of root extension, as occurs in heavier soils, can increase the extent of pre-emergence damping-off (Barbetti and You, unpublished). Establishing adequate seedling density is critical for early-season subterranean clover production as it closely correlates with seedling density (Donald, 1951), and pre-emergence damping-off also reduces persistence of subterranean forages, the latter leading to increased weedy content of forages (Barbetti et al., 2006b; Jones and Barbetti, 2012). The prevalent decline of subterranean clover forages and their failure to persist long term has led to severe decreases in both the capacity to carry livestock and the overall whole-farm sustainability and profitability across southern Australia (Barbetti et al., 1986b; Nichols et al., 2014; You and Barbetti, 2017b).

In relation to tap and lateral root disease, there were significant interactions between temperature, soil, moisture, and nutrition, and for tap root disease between soil, moisture, nutrition and variety and lateral root disease between soil and variety. That boosted regression trees found similar relative influence for both tap root and lateral root disease index of soil, temperature and variety was expected, as tap and lateral root disease severities are strongly and positively correlated across different situations, environments and even varieties in other studies (e.g., Barbetti and MacNish, 1984; Wong et al., 1986a). There have been other studies with subterranean clover to relate environmental factors to severity of damping-off and/or root disease, but, except for a study involving *P. clandestina* (You and Barbetti, 2017b) or *R. solani* (You and Barbetti, 2017a), these involved a single or only a very small number of explanatory variables. For example, associating higher and more frequent autumn seasonal rainfall with a particularly severe root disease year, compared with other years of less frequent and overall rainfall when there was a much lower severity of root disease (MacNish et al., 1976). In the current study, there were higher levels of tap and lateral root rot disease in high moisture treatments, to be expected as wet soil conditions strongly encourage attack by oomycetes like *Pythium* or *Phytophthora* spp. on germinating seedlings and/or root systems of surviving plants (You and Barbetti, 2017b). Even brief periods of soil saturation from flooding promote infection of roots by *Pythium* spp. because they thrive and produce massive numbers of motile zoospores under such conditions (Yanar et al., 1997; Li et al., 2015). Wong et al. (1984), however, examined the effects of temperature and moisture on *P. irregulare*, showing that it was specific temperature/moisture combinations that resulted in the most severe root disease (e.g., flooding across 10, 15, 20, and 25°C). That there was an effect of nutrition level on root disease was not unexpected, as nutrient stress enhances the susceptibility of plants to disease (Graham, 1983). Previous studies by O’Rourke et al. (2012) highlighted how application of a full range of plant nutrients to field soils lowered tap and lateral root disease severity in subterranean clover by roughly 45 and 32%, respectively; and that application of either K or N alone reduced tap root disease severity by >30%. In addition, application of a complete fertilizer (200 kg ha^-1^ of superphosphate containing Cu, Zn, and Mo, plus 50 kg ha^-1^ of potash) in field trials across southern Australia in 2015 and 2016, increased productivity of subterranean clover in stands severely affected by soilborne root disease by up to 1.5-fold (You and Barbetti, unpublished data). Taken together, improved nutrition likely offer considerable potential for alleviating the impact of soilborne root pathogens such as *P. irregulare*. However, the relative proximity of placement of added nutrition to germinating seeds and seedling roots will likely regulate the extent to which plants uptake and utilize any additional nutrients provided and, as such, determine any influence on levels of pre- and post-emergence damping-off and severity of root rot of surviving plants.

Variety was also an important factor. In decision trees, least disease was for Seaton Park and Woogenellup in sand-based soil under higher and medium temperatures and at higher moisture for tap roots. A similar pattern was found for least lateral root disease for Seaton Park and Woogenellup grown in sand-based soil under high moisture and medium temperature. The most severe tap root disease was in loam soil under low moisture and high nutrition, but for lateral root disease it was most severe under low moisture for Riverina. Heat maps showed greatest tap and lateral root disease for loam soil, particularly for tap than compared with lateral root disease. Variety of subterranean clover significantly affected severity of both tap and lateral root disease and also pre-emergence damping-off. In relation to strong interactions in the current study between variety with soil type, temperature and moisture, it is noteworthy that Seaton Park and Woogenellup had least tap and lateral root disease. This was a somewhat surprising outcome for Woogenellup as it is known to be very susceptible to Pythium root rot (Wong et al., 1984, 1985a, Wong et al., 1986a; You et al., 2005a), but it is known to be productive despite presence of *P. irregulare* providing conditions for rapid growth are present such as warmer spring temperatures. However, improved varietal host resistance remains the focus if root disease severity is to be reduced and productivity of subterranean clover forages is to be increased, particularly when environmental conditions are conducive for development of severe disease (Barbetti et al., 2007; Nichols et al., 2014; You and Barbetti, 2017b). Recent screening of subterranean clover varieties against both individual soilborne pathogens and against a combination of pathogens including *P. irregulare, A. trifolii, R. solani* and multiple races of *P. clandestina*, highlighted both strong and effective individual ‘resistances’ and general ‘field tolerances’ (You and Barbetti, unpublished data). These general ‘field tolerances’ are particularly valuable, particularly as fungicides, while sometimes effective seed treatments (Barbetti et al., 1987a) often show variable efficacy in the presence of multiple soilborne pathogens (Barbetti et al., 1987b) and particularly when applied to long-standing forage stands even as soil drenches (Barbetti et al., 1987b).

As previous studies with *P. irregulare* had shown strong negative relationship between root disease severity and productivity of subterranean clover as expressed by shoot dry weight (e.g., Barbetti and MacNish, 1978; Barbetti, 1984a,b; Barbetti et al., 1986b; Wong et al., 1984), it was not surprising that *P. irregulare* significantly reduced shoot weight (i.e., productivity) in the current study. Modeling found a complex relationship between dried shoot weights of plants infested with *P. irregulare* and explanatory variables, linear modeling highlighted significant interaction between all explanatory variables in terms of both dry shoot and dry root weight, and BRTs provided further support for the influence of all explanatory variables in differences in root weights, the latter showing that soil type most influenced dry weights, followed by temperature, nutrition, variety, and moisture. The most productive plants, with the greatest dry shoot weight, were planted in sand-based soil, under high or medium temperatures, and plants with the greatest dry root weight were similarly grown in sand-based, but with high moisture and high nutrition. Similarly, heat maps showed a clear increase in dry shoot and root productivity for plants grown in sand-based, in medium or high temperatures; but least productivity was for loam soils, particularly under low temperatures. The findings in relation to greater productivity under warmer temperatures are in line expectations, as in the presence of *P. irregulare* Wong et al. (1984) showed largest subterranean clover shoots were produced at the warmer temperature conditions (e.g., 25°C) compared with cooler temperatures (e.g., 10°C). O’Rourke et al. (2012) highlighted the scope to utilize applications of one or more nutrients as part of a more comprehensive approach to manage root disease, as discussed above, and also to increase size of subterranean clover root systems and consequent shoot growth, particularly in the commonly occurring situations across southern Australia where soils are naturally deficient in one or more critical nutrients. Improving nutrition offers opportunities to alleviate the adverse impacts of *P. irregulare* and increase productivity of subterranean forages.

*Pythium* spp. such as *P. irregulare* cause devastating root disease and/or damping-off worldwide across a wide range of economically important crops (Bala et al., 2010), are considered the overriding reason for declining production of critical food crops such as common bean (*Phaseolus vulgaris*), and remain a principal constraint to many crops worldwide (Abawi and Widmer, 2000). Further, *P. irregulare* is the most common *Pythium* species present in soils (Hendrix and Campbell, 1970). The greatest adverse impact of soilborne pathogens like *P. irregulare* coincides with the time of the naturally occurring feed shortage over the autumn–winter period. Severe disease markedly curtails the autumn–winter biomass in regenerating subterranean clover forage stands (You and Barbetti, 2017a,b). Hence, the need to determine and understand the role of environmental factors in Pythium damping-off and root disease. It is clear that variable and fluctuating temperature and moisture conditions, normal for annual subterranean clover forages across southern Australia (Barbetti and You, 2014) and as demonstrated in the current study, determine the severity and impact of soilborne disease epidemics. However, faced with warming temperatures across the southern Australian forage and cropping zones (Barbetti et al., 2012; Jones and Barbetti, 2012), not only will the relative importance of the different environmental factors likely change in association with these future climate scenarios, but the relative importance of pathogens, including soilborne pathogens such as *P. irregulare*, will also likely alter (Chakraborty et al., 1998), possibly becoming less severe under future predicted warmer growing season temperatures (Barbetti et al., 2012; Jones and Barbetti, 2012).

It is evident from the current study that areas of sand-based soil will have greater emergence, less disease and greatest persistence and productivity and these may be productive irrespective of variety grown. In contrast, the most diseased, least persistent and least productive subterranean clover forages will likely be for heavier soils (e.g., loam soil) and when temperatures are low. However, this situation may be altered following ‘breaking up’ and ‘loosening’ the soil structure following cultivation (Barbetti and MacNish, 1984; You et al., 2017). There were higher levels of tap and lateral root rot disease in higher moisture situations. Where conditions are conducive for severe disease, affected forages would require management utilizing a combination of relatively expensive cultural practices [e.g., cultivation (Barbetti and MacNish, 1984; You et al., 2017)] in conjunction with reseeding with more disease resistant varieties where they are available (Barbetti et al., 2006a,b, 2007). Improved host resistance offers the most cost-effective and long term means to limit losses where severe Pythium damping-off and root disease occurs, and would provide certainty of production even under seasonal variations in moisture, temperature, nutrition that favor severe Pythium damping-off and root disease. However, unfortunately, effective host resistance against *P. irregulare* is rare within Australian subterranean clover varieties, with only Karridale showing strong resistance and Dinninup, Enfield, Mt Barker and Urana showing only moderate resistance (Nichols et al., 2014). Despite this, the existence of high level resistance in some breeding lines (You et al., 2005a), the existence of some varieties with resistance to two or more of the main pathogens in the soilborne pathogen complex (You et al., 2005b), and the recent discovery of the first effective field tolerance against the entire soilborne disease complex (You and Barbetti, unpublished data) offer opportunity for Pythium damping-off and root disease to be cost-effectively managed in future for the first time.

In terms of future studies, in comparison to studies with an individual pathogen such as *P. irregulare* used in the current study or *R. solani* (You and Barbetti, 2017a) or *P. clandestina* (You and Barbetti, 2017b), there remains opportunity to explore more complex interactions of ‘environmental factors’ with multiple pathogen complexes. However, understanding synergisms between microbial pathogens is critical to understanding such pathogen complexes (Lamichhane and Venturi, 2015; Lamichhane et al., 2017). Of particular relevance is the recent studies by Foster et al. (2017) who highlighted the existence of widespread ‘natural synergistic associations in the field between *Rhizoctonia* and *Pythium* spp., *Pythium* and *Fusarium* spp., *Pythium* spp. and *A. trifolii*, and *P. clandestina* and *A. trifolii*’ in association with damping-off and root disease of subterranean clover forages. Further, there remain other ‘environmental factors’ not considered in the current study. For example, Burdon and Chilvers (1975) previously analyzed and modeled the impact of planting density on epidemics of damping-off disease caused by *P. irregulare*, highlighting how planting density is also an important factor affecting epidemiology of damping-off or root diseases caused by soil-borne pathogens. The current studies are but the first stage in revealing and elucidating the complex environmental-pathogen interactions associated with damping-off and root disease in subterranean clover forages.

## Author Contributions

MY and MB designed the study MY conducted the environment × disease studies and conducted initial analyses and data presentation. KR and MR undertook the modeling and its interpretations. MB and MY wrote the first draft of the paper. All authors contributed to revisions.

## Conflict of Interest Statement

The authors declare that the research was conducted in the absence of any commercial or financial relationships that could be construed as a potential conflict of interest.
