# Supplementary material for: Modeling Effects of Temperature, Soil, Moisture, Nutrition and Variety As Determinants of Severity of Pythium Damping-Off and Root Disease in Subterranean Clover
Source: Front Microbiol. 2017 Nov 14;8:2223. doi: 10.3389/fmicb.2017.02223 (PMC5694439; doi:10.3389/fmicb.2017.02223)
Supplement: Supplementary file 1 [file Data_Sheet_1.PDF]

Supplementary Material

Modelling Effects of Temperature, Soil, Moisture, Nutrition and Variety as Determinants of Severity of Pythium Damping-off and Root Disease in Subterranean Clover

Ming Pei You, Kelly Rensing, Michael Renton and Martin J. Barbetti*

*** Correspondence:** Corresponding Author: martin.barbetti@uwa.edu.au

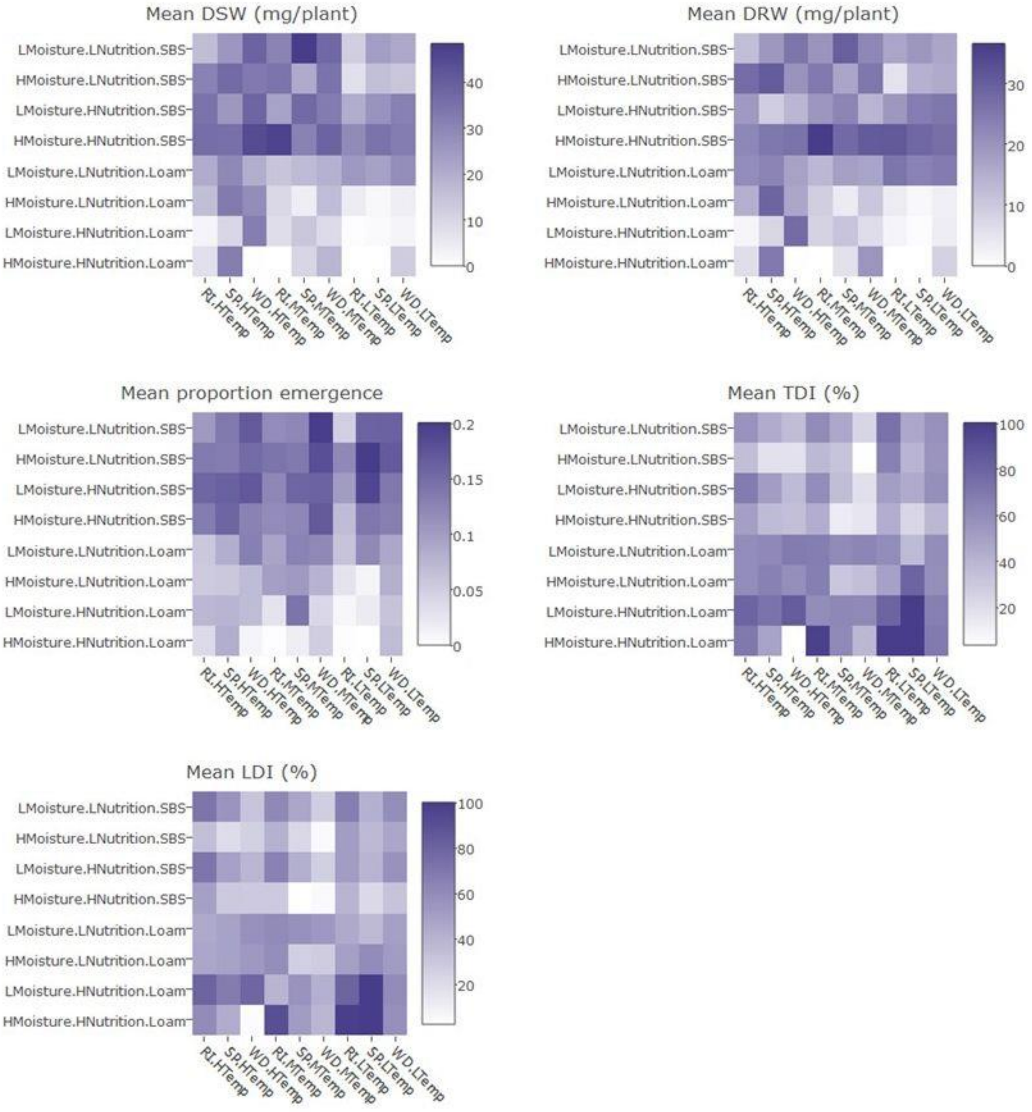

Supplementary Figure 1. Heat maps of mean predictions from linear modelling of the effects of environmental variables on the dried shoot weight (DSW), dried root weight (DRW), emergence, and tap root and lateral root disease indices (TDI, LDI) of three subterranean clover (*Trifolium subterraneum*) varieties inoculated with Pythium root-rot (*Pythium irregulare*) in a controlled experiment and grown under combinations of high (H) and low (L) moisture and nutrition and in sand-based soil (SBS) or loam soil (Loam).

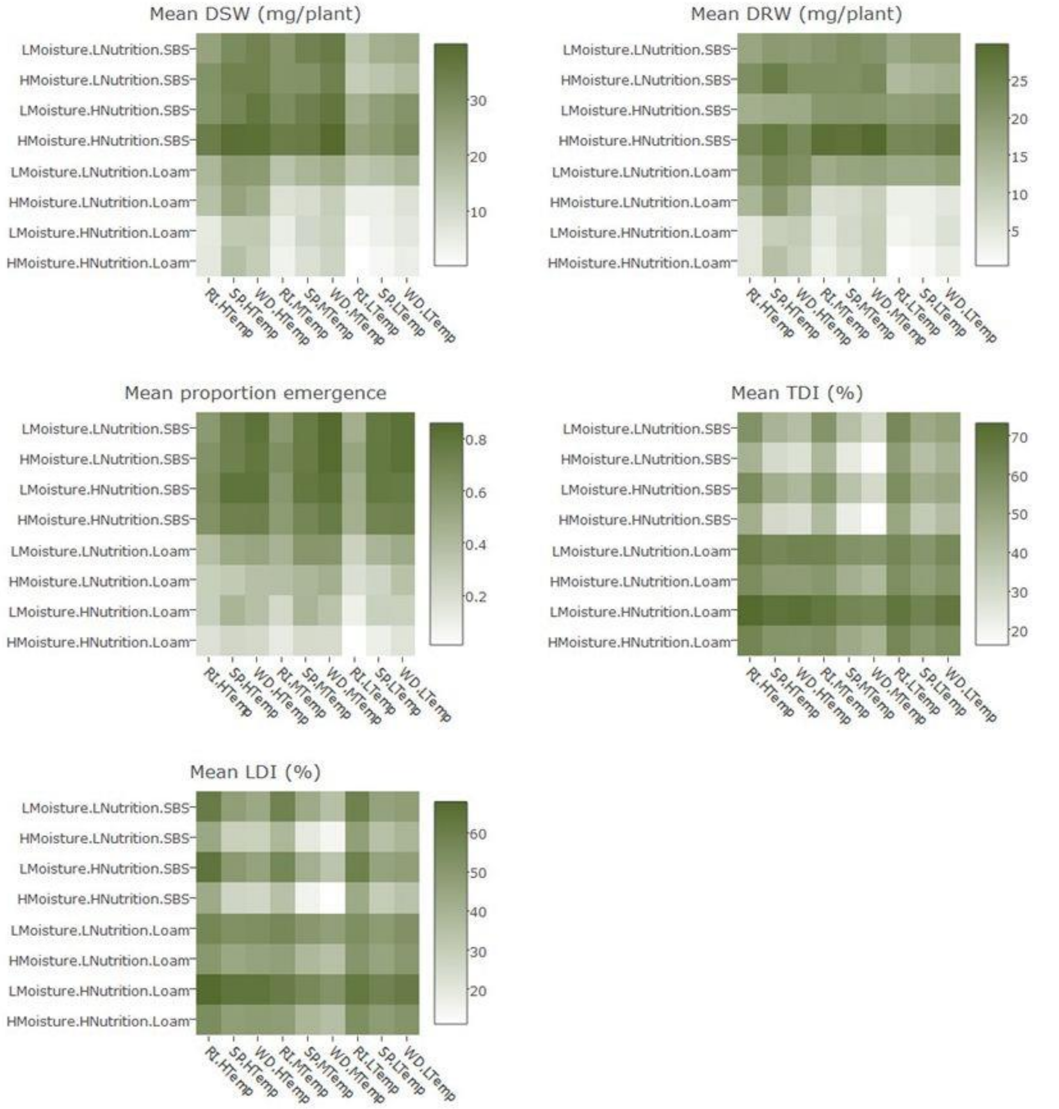

Supplementary Figure 2. Heat maps of mean predictions from boosted regression trees modelling of the effects of environmental variables on the dried shoot weight (DSW), dried root weight (DRW), emergence, and tap root and lateral root disease indices (TDI, LDI) of three subterranean clover (*Trifolium subterraneum*) varieties inoculated with Pythium root-rot (*Pythium irregulare*) in a controlled experiment and grown under combinations of high (H) and low (L) moisture and nutrition and in sand-based soil (SBS) or loam soil (Loam).

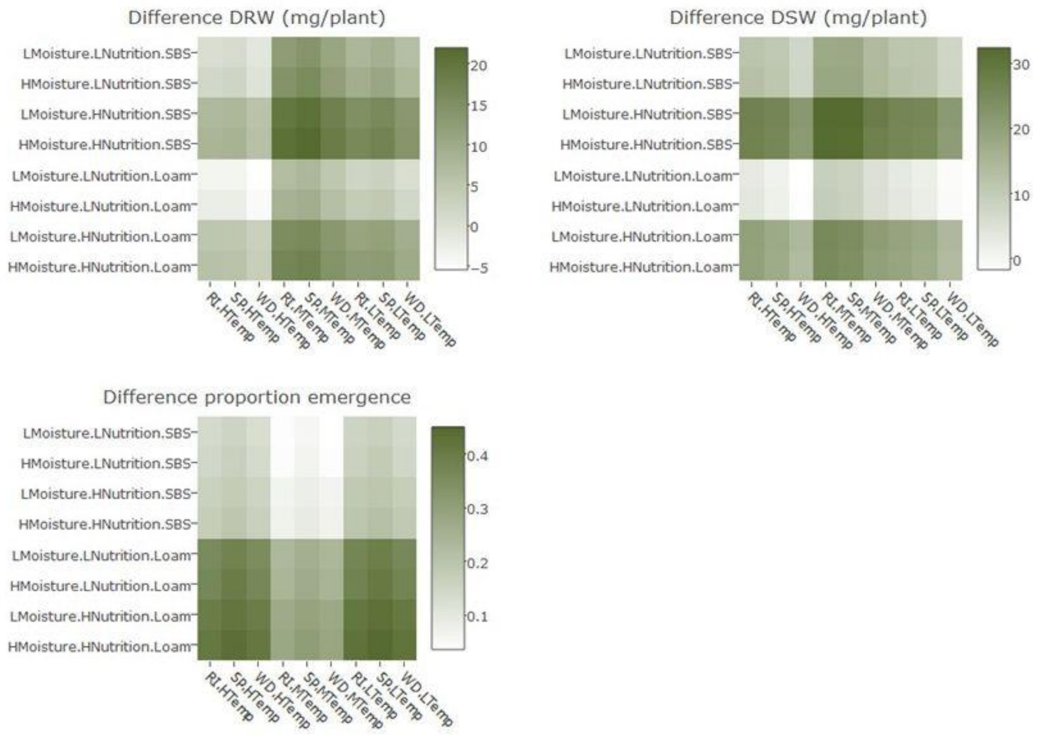

Supplementary Figure 3. Heat maps of mean predictions from boosted regression trees modelling the difference between mean dried shoot weight (DSW), dried root weight (DRW) and emergence of three subterranean clover (*Trifolium subterraneum*) varieties inoculated with Pythium root-rot (*Pythium irregulare*) and emergence of control plants and grown under combinations of high (H) and low (L) moisture and nutrition and in sand-based soil (SBS) or loam soil (Loam).
